# Natrium Alginate and Graphene Nanoplatelets-Based Efficient Material for Resveratrol Delivery

**DOI:** 10.3390/gels11010015

**Published:** 2024-12-27

**Authors:** Cristina Mormile, Ocsana Opriș, Stefano Bellucci, Ildiko Lung, Irina Kacso, Alexandru Turza, Adina Stegarescu, Septimiu Tripon, Maria-Loredana Soran, Ioana Bâldea

**Affiliations:** 1 National Institute for Research and Development of Isotopic and Molecular Technologies, 67-103 Donat, 400293 Cluj-Napoca, Romania; mormile@rait88.com (C.M.); ocsana.opris@itim-cj.ro (O.O.); ildiko.lung@itim-cj.ro (I.L.); irina.kacso@itim-cj.ro (I.K.); alexandru.turza@itim-cj.ro (A.T.); adina.stegarescu@itim-cj.ro (A.S.); septimiu.tripon@itim-cj.ro (S.T.); 2 R.A.I.T. 88 S.R.L, Via Pieve Torina 64/66, 00156 Rome, Italy; stefano.bellucci@lnf.infn.it; 3 Department of Chemistry, Sapienza University of Rome, Piazzale Aldo Moro 5, 00185 Rome, Italy; 4 INFN-Laboratori Nazionali di Frascati, Via E. Fermi 54, 00044 Frascati, Italy; 5 Electron Microscopy Center, Babes-Bolyai University, 400006 Cluj-Napoca, Romania; 6 Department of Physiology, Iuliu Haţieganu University of Medicine and Pharmacy, Clinicilor 1, 400006 Cluj-Napoca, Romania; baldeaioana@gmail.com

**Keywords:** drug delivery, graphene, resveratrol, alginate, kinetic analysis

## Abstract

In this study, alginate-based composite beads were developed for the delivery of resveratrol, a compound with therapeutic potential. Two formulations were prepared: one with sodium alginate and resveratrol (AR) and another incorporating graphene nanoplatelets (AGR) to improve drug release control. The beads were formed by exploiting alginate’s ability to gel via ionic cross-linking. For the AGR formulation, sodium alginate was dissolved in water, and graphene was dispersed in isopropyl alcohol to achieve smaller flakes. Resveratrol was dissolved in an ethanol/water mixture and added to the graphene dispersion; the resulting solution was mixed with the alginate one. For the AR formulation, the resveratrol solution was mixed directly with the alginate solution. Both formulations were introduced into a calcium chloride solution to form the beads. The release of resveratrol was studied in phosphate-buffered saline at different pH values. Results showed that the presence of graphene in the AGR sample increased drug release, particularly at pH 6.8, indicating a pH-driven release mechanism. Kinetic analysis revealed that the Higuchi model best describes the release mechanism. Finally, cytotoxicity tests showed the biocompatibility of the system in normal human cells. These findings suggest that graphene-enhanced alginate matrices have significant potential for controlled drug delivery applications.

## 1. Introduction

Diseases significantly impact populations, driving the need for advanced therapeutic strategies. Modern drug delivery technology began evolving in 1952 with the introduction of the first sustained-release capsule [1]. Since then, continuous research has enhanced the field. Drug delivery systems are critical because direct administration of active pharmaceutical ingredients (APIs) is often impractical due to issues such as irritation at the administration site [2,3], environmental degradation of APIs (e.g., exposure to pH, temperature, light, and moisture) [4], and delivery inefficiencies.

The term “system” encompasses various drug formulations, including tablets, capsules, and solutions. These formulations release APIs either in a controlled or immediate manner and are classified based on their release mode. Their therapeutic effectiveness largely depends on these characteristics [1,5]. ”Release” refers to the movement of a specific component from its formulation into the external environment. This release can occur in several forms:

- Immediate release: APIs are released immediately after administration.
- Modified release: Release occurs at an unspecified or extended time.
- Targeted release: APIs are directed toward specific body sites.

The most valuable type is controlled release, which ensures a predetermined release rate, maintaining an optimal drug concentration at the target site [6]. Determining the best release mechanism involves studying release kinetics through linear and non-linear regression analyses [7]. Dissolution profiles are then developed based on the selected model parameters.

As these systems are formulations, they typically consist of two main components: the drug and the carrier. Carriers vary widely, including polymers, liposomes, and nucleic acids, and are tailored to optimize performance [8]. Polymers, especially biopolymers, are extensively studied for their advantageous properties, such as biocompatibility, biodegradability, availability, and favorable physicochemical characteristics [7].

This study focuses on sodium alginate (NaAlg), a biopolymer composed of β-D-mannuronic acid (M) and its C-5 epimer, L-guluronic acid (G) [9]. NaAlg forms gels through cross-linking with divalent cations and is widely used in the pharmaceutical industry as an excipient for emulsification, stabilization, thickening, and flavoring [10]. Its high biocompatibility, biodegradability, and ability to incorporate and release proteins have led to its application in various medical fields, including wound healing [11], tissue regeneration [11,12], and drug delivery [9]. Alginate-based drug delivery methods include hydrogels, tablets, capsules, liposomes, nanoparticles, and microspheres [13,14]. These gels are stable between 0 °C and 100 °C and dissociate in acidic environments. To ensure homogeneity, gel production requires precise control of experimental conditions such as alginate concentration, temperature, and cation type [14].

To enhance drug delivery efficiency, hybrid composites are often employed. These respond to external stimuli, enabling targeted API release [15]. Among nanoscale fillers, graphene and its derivatives have drawn significant attention. Graphene’s large hydrophobic surface stabilizes drugs, while its conductivity and mechanical properties can modulate release [16]. However, graphene’s cytotoxicity—determined by flake size and C/O ratios—remains a key consideration [17,18]. Larger ratios result in lower cytotoxicity [19].

Stabilizing APIs facilitates targeted drug delivery, reducing systemic side effects [20,21]. One extensively studied API is resveratrol, known for its applications in treating neurological disorders [22], anti-inflammatory properties, hepatoprotection, cardio- and neuroprotection, anti-cancer effects, anti-aging benefits, and diabetes prevention [23]. However, resveratrol’s systemic bioavailability is limited by extensive metabolism, poor solubility and permeability, light-induced isomerization, and auto-oxidation [24]. Encapsulation significantly improves its bioavailability, though this strategy remains underexplored with carriers like alginate and graphene [23].

This study aimed to develop a drug delivery system combining sodium alginate, graphene, and resveratrol. This system was compared with a sodium alginate-resveratrol system to assess graphene’s potential to enhance controlled release. Release tests were performed at 37 °C and pH values of 7.4 and 6.8, simulating physiological conditions and exploring pH-dependent release in the graphene-containing formulation. Moreover, cytotoxicity tests were conducted in normal human cells in vitro to assess the biocompatibility of the system. The novelty of this work lies in the incorporation of graphene nanoplatelets, prepared via microwave synthesis, into the bead preparation system.

## 2. Results and Discussion

### 2.1. Beads Formation and Characterization

**Figure 1:**
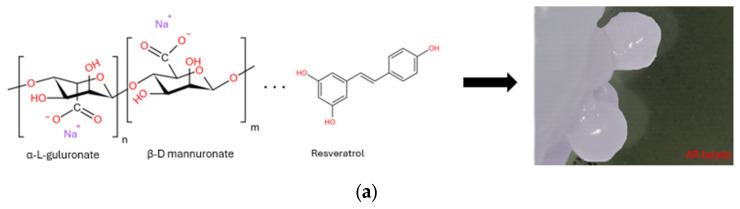

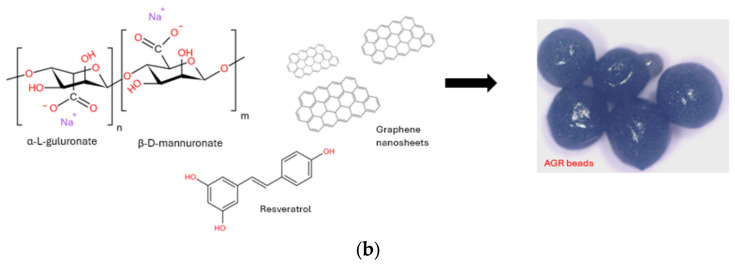
AR (**a**) and AGR (**b**) samples.

Sodium alginate (NaAlg) is a plant-based polymer extracted from various brown algae species such as *Laminaria*, *Macrocystis*, *Sargassum*, *Ascophyllum*, *Lessonia*, *Ecklonia*, and *Durvillea* [25]. It belongs to the polysaccharide family and is composed of two monomers: β-(1-4)-D-mannuronic acid (M) and α-L-guluronic acid (G). The ratio between these two monomers varies depending on different factors, which in turn can influence the properties of the polymer [26]. Thanks to its valuable characteristics, alginate has been widely studied and used in the pharmaceutical field. Several drug delivery strategies using include hydrogels, tablets, capsules, liposomes, nanoparticles, and microspheres [9,27]. The first step in synthesizing the beads involves dissolving alginate powder in water for about 20 min until it is completely dissolved, forming a jelly like liquid. Calcium alginate beads are then produced by slowly adding the sodium alginate solution into a calcium chloride solution. Once all the solution is added, the system is left to rest for 20 min to stabilize. Two samples were produced: AR (alginate + resveratrol) and AGR (alginate + graphene + resveratrol) (Figure 1). The alginate matrix formed is typically permeable, meaning that controlling the release might not be optimal [28], particularly for soluble drugs. Since resveratrol is a partially water-soluble drug, graphene was incorporated into the composite to enhance the polymer’s properties. For the AGR sample, a graphene dispersion was added to study the interactions between graphene and resveratrol, which may improve the control over drug release. Graphene is a planar sheet composed of six-membered carbon rings with sp² hybridization. Due to its exceptional physicochemical, optical, electrical, and mechanical properties, it has been used in a wide range of applications, including energy storage, sensors, and biological and biomedical fields [29]. In the latter, graphene’s most valuable feature is its layered structure, which facilitates better drug loading [30]. To address graphene’s low dispersibility in water and avoid cytotoxicity caused by aggregation, graphene is often mixed with polymers. This combination leverages both covalent and non-covalent interactions, enhancing the properties of all components within the system [19]. The presence of graphene is expected to improve drug release control over time, as its incorporation creates new interactions that can modify how the drug is released. In particular, resveratrol, which contains aromatic rings, could form π-π stacking interactions with graphene, promoting the absorption of the drug onto the graphene surface. This enhances both the stability of the drug and its integration with alginate.

**Figure 2:**
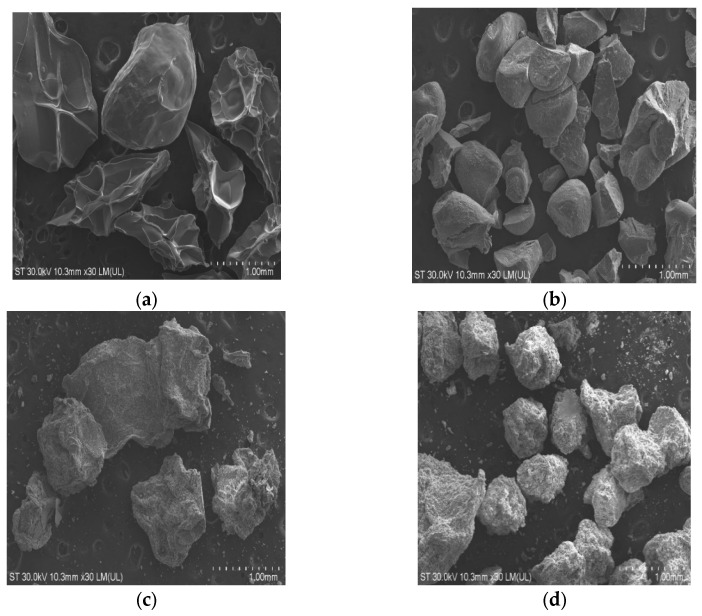
SEM images of (**a**) Alginate beads; (**b**) Alginate and graphene beads; (**c**) Alginate and resveratrol beads; (**d**) Alginate, graphene, and resveratrol beads.

To confirm the successful incorporation of graphene and resveratrol into the alginate beads, several analyses were performed, including infrared spectroscopy and SEM imaging. Figure 2 shows the SEM images of the A (alginate), AG (alginate + graphene), AR (alginate + resveratrol), and AGR samples. Sample A exhibits a smoother surface, which becomes less homogeneous after the addition of resveratrol (R) and graphene (G), due to the integration of these compounds into the polymer matrix and their deposition on the polymer surface. In panel (d), it is clearly visible that the amount of resveratrol on the surface of the beads is greater than in panel (c), likely due to the presence of graphene, which traps the drug through hydrophobic interactions and π-π stacking.

**Figure 3:**
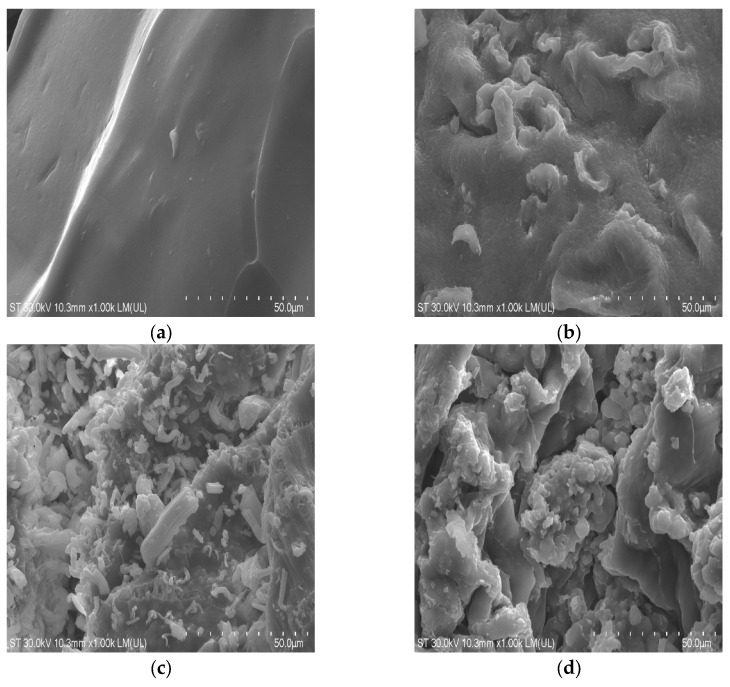
SEM images of: (**a**) Alginate beads; (**b**) Alginate and graphene beads; (**c**) Alginate and resveratrol beads; (**d**) Alginate, graphene, and resveratrol beads.

Upon closer examination of the bead surfaces, the differences between the samples become more apparent after each component is added (Figure 3). Sample A displays a very smooth surface. In contrast, the surface of sample AG becomes rougher due to the integration of graphene nanoplatelets, which position themselves between the polymer chains and cause surface deformation. The AR (alginate + resveratrol) sample shows both the presence of resveratrol, as previously reported in the literature [31], on the surface of the beads, as well as within the beads themselves. This is evident from the external surface being less smooth compared to that of sample A. Finally, in the last image, the craters on the surface, attributed to the presence of both graphene and resveratrol molecules, are clearly visible, confirming the incorporation of both components into the composite.

**Figure 4:**
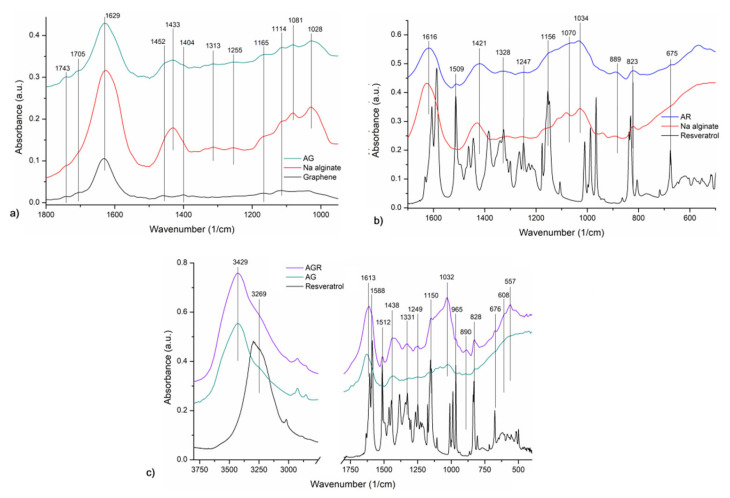
FTIR spectra of: (**a**) graphene Na alginate and AG spectral domain 1800–950 cm^−1^; (**b**) resveratrol, Na alginate, and AR, spectral domain 1700–500 cm^−1^ s (**c**) resveratrol, AG, and AGR, spectral domain 3800–400 cm^−1^, break region 2750–1800 cm^−1^.

Moreover, FTIR analysis was carried out on the samples, confirming the expected results. The characteristic vibrational bands of Na alginate can be identified as follows (Figure 4a): stretching vibrations of -OH groups at 3425 cm^−1^, asymm. and symmetric stretching of CH_2_ groups at 2927 and 2856 cm^−1^, and COO- at 1626 and 1431 cm^−1^, stretching vibrations of C–O bonds at 1312 cm^−1^, C–C bonds at 1168 sh and 1114 sh cm^−1^, stretching vibrations of C-O and C–O–C groups from the mannuronic and guluronic units at 1081 and 1028 cm^−1^, respectively [32], stretching of C–O groups from pyranosyl ring with C–C–H and C–O–H deformation contributions at 939 cm^−1^, vibration of C-O groups in α-configuration of the glucuronic units at 820 cm^−1^ [16,33,34]. The FTIR spectrum of graphene (Figure 4a) showed the characteristic vibrational bands of graphene and starting graphite: O-H bending and C=C stretching vibrations at 3433 and 1631 cm^−1^ [34,35], assym. and symm. vibration of C-H bonds at 2923 and 2854 cm^−1^, low-intensity bands of C=O, C-OH, and C-O groups vibrations at 1705 sh, 1526, 1458, 1399, 1169, 1115, and 1036 cm^−1^, and low-intensity skeletal vibration of the graphene sheets at 1545 cm^−1^ [36].

The most important vibrational bands of resveratrol (Figure 4b) can be assigned like this: stretching vibrations of –OH, as broadband with maxima at 3297 cm^−1^, stretching vibrations of the aromatic −C=C− at 1606, 1513 and 1444 cm^−1^, of the −C−O− bonds, of olefinic −C−C−, as well as trans olefinic −C=C− bending vibrations at 1587, 1154, 966 and 831 cm^−1^, respectively, corresponding with literature data [37,38].

In the spectrum of the AG sample (Figure 4a), both the characteristic vibration bands of graphene and those of Na alginate can be identified with small shifts. Thus, the vibrations of COO-, C=O, C-OH, and C-O groups of graphene at 1629, 1452 sh, and 1404 sh cm^−1^ and the Na alginate’s vibrations at 1430, 1165 sh, 1114, 1081, and 1028 cm^−1^. The spectrum of the AR sample (Figure 4b) showed the characteristic vibration bands of both Na alginate and resveratrol, as follows: in the spectral range 3700–3000 cm^−1^ broadband corresponding to stretching vibrations of -OH with maxima at 3426 cm^−1^, and a shoulder at 3289 sh cm^−1^, respectively. The alginate’s COO- stretching vibrations were shifted at 1616 and 1421 cm^−1^, stretching vibrations of C-O and C–O–C groups at 1034 cm^−1^. The stretching vibrations of aromatic −C=C− from resveratrol were shifted at 1509 cm^−1^, and bending of trans olefinic −C=C− are found at 1156 cm^−1^, as a shoulder, and at 823 cm^−1^. In the spectrum of the AGR sample (Figure 4c), the presence of resveratrol is evident through the appearance of characteristic bands, which can be identified as new peaks compared to those of AG or as an increase in intensity due to overlapping bands with minor shifts: These include the following bands: 3269 cm^−1^ as a shoulder, 1588 sh, 1512, 1438, 1426, 1331, 1249, 1150, 965, 828, and 676 cm^−1^.

### 2.2. In Vitro Drug Release

**Figure 5:**
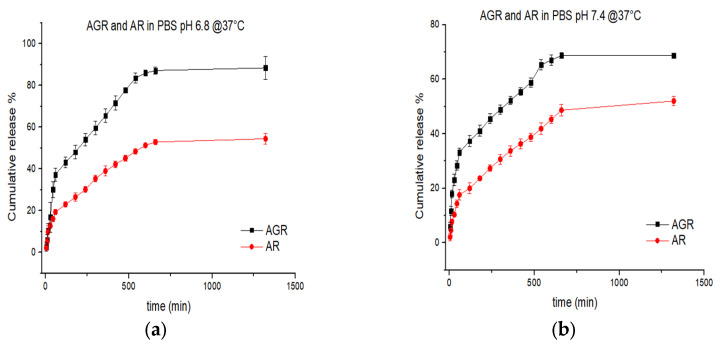
Release pattern of the samples: (**a**) AGR and AR in PBS pH 6.8; (**b**) AGR and AR in PBS pH 7.4.

The drug release tests were conducted at 37 °C for both AR and AGR samples at two different pH values to compare resveratrol behavior under different environmental conditions and to investigate the influence of pH on the release system. The entire batch was placed in 20 mL of PBS at pH 6.8 and 7.4. To prevent resveratrol degradation, the test tubes were covered with aluminum foil. Aliquots of the medium were taken at the following time points: 5, 10, 15, 30, 45, 60, 120, 180, 240, 300, 360, 420, 480, 540, 600, 720, and 1440 min. After each aliquot was taken, an equal volume of fresh medium was added to maintain sink conditions for the experiment. The aliquots were analyzed using UV-Vis spectrophotometry, and the absorbance values obtained were interpolated using calibration curves to determine the exact resveratrol concentration in the solution at each sampling time. The cumulative release percentage is shown in Figure 5. At pH 6.8, the AR system released 54% of the loaded drug after 24 h, while the AGR system released 88% of the total drug. At pH 7.4, AR released 52%, and AGR released 66% of the total drug.

**Figure 6:**
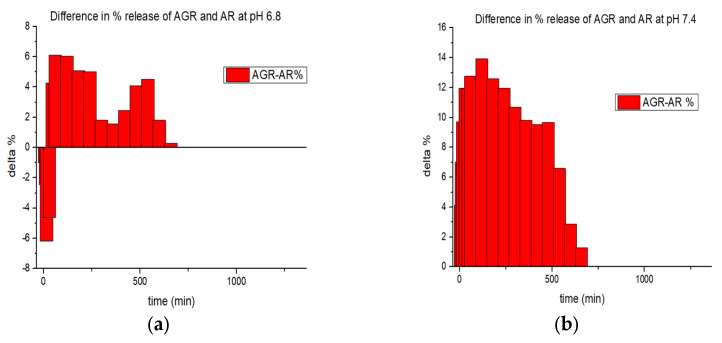
Difference between AGR and AR of drug released at every sampling point. (**a**) Results at pH 6.8; (**b**) Results at pH 7.4.

In both experiments, the amount of resveratrol released by AGR was greater than that released by AR. This difference can be attributed to the washing phase with distilled water, during which the AR sample experiences a loss of resveratrol due to the high permeability of the alginate molecules. In contrast, the presence of graphene in AGR, along with the hydrophobic interactions it induces, helps retain the drug within the composite matrix, preventing its loss during the preparation phase. Furthermore, at both pH values, the cumulative release percentage of AR remains constant, whereas for AGR, there is a 20% difference, suggesting that the graphene-based system is pH-dependent. This observation also supports the hypothesis that any potential resveratrol degradation in the medium does not significantly affect the final results. Figure 6 highlights the difference in drug release at each sampling point, providing a deeper insight into the release system’s characteristics. At pH 6.8, the delta is negative during the first hours, indicating that more drug is released from the AR system than from the AGR system. However, after the initial burst release, the trend reverses, with AGR releasing more drug than AR for the remainder of the experiment. At pH 7.4, the delta remains positive throughout the entire experiment, meaning that the AGR sample consistently delivers a higher amount of the drug. The pH dependence could be attributed to the fact that, in the AR samples, resveratrol primarily interacts with the polymer matrix through hydrogen bonds, whereas in the AGR samples, hydrophobic interactions are more likely to occur. Moreover, hydrogen bonds are stronger at neutral pH, so at acidic pH, the AR sample releases resveratrol at a faster rate than the AGR sample. However, at pH 7, the AGR sample releases the drug more rapidly due to the stronger interactions in the sample at this pH [39].

To better understand these results, the regression coefficients and kinetic parameters of the tests were calculated for several models, including zero-order, first-order, Higuchi, Hixson–Crowell, and Korsmeyer–Peppas. The parameters for the entire experiment are summarized in Table 1, where the best-fitting release model for all the samples was found to be the Higuchi model. The Higuchi model is based on the assumption of instantaneous drug dissolution and a pseudo-steady-state drug release. The equation for the release mechanism from a spherical matrix immersed in a perfect sink is [40,41]:

(1) $$\mathrm{Fraction}\;\mathrm{of}\;\mathrm{released}\;\mathrm{drugs}=1-{\left(\eta^{*}\right)}^{3}-\int_{\eta^{*}}^{1}\varphi \cdot 3\eta^{2}\cdot d\eta$$

where η is the dimensionless radius of the sphere and η^*^ is the dimensionless moving front. After all the necessary simplifications, the final equation is [42]:

(2) $$Q_{t}=Q_{0}+K_{H}t^{1/2}$$

where $K_{H}$ is the Higuchi dissolution constant, which holds significant importance for understanding the release mechanism in a physically realistic context. When a drug release system fits the Higuchi model, it indicates that the drug molecules are primarily released through controlled diffusion, with swelling playing a limited role in the release process [42]. Moreover, the diffusion process follows Fick’s law and is dependent on the square root of time, with secondary release mechanisms such as dissolution, partitioning, and swelling contributing to a lesser extent [6].

The value of the Higuchi dissolution constant provides information on the rate and efficiency with which the drug is released from the matrix over time. It is closely related to the solubility of the drug and the porosity of the matrix. When K_H_ has higher values, the drug is released more rapidly into the system [43], the values for the experiment show that for AR, the release is slightly faster than for AGR. Although the R^2^ value of the Higuchi model is higher, the Korsmeyer–Peppas model should also be considered due to the similarity in the values of the coefficients. The Korsmeyer–Peppas model was originally developed to describe drug release from polymeric systems, such as hydrogels [44]. This semi-empirical model establishes an exponential relationship between release and time and is particularly useful when more than one release mechanism is involved.

In this case, since both regression coefficients are around 0.9, the Korsmeyer–Peppas model suggests a non-Fickian diffusion process, which contrasts with the behavior observed in the Higuchi model. This anomalous behavior can be explained by the onset of a new diffusion mechanism: the erosion of the beads’ surface in solution after the first 6 h. This hypothesis is supported by the observation that when plotting the release data for only the first six hours (as shown in Table 2), the regression coefficient for the Higuchi model fits the data much better than the Korsmeyer–Peppas model [45].

Erosion could be an important factor in achieving drug release even after 24 h; however, it may be more challenging to control the amount of API released. Therefore, future research should consider conducting drug release tests over a longer period, beyond 24 h. In the literature, there are no other examples of drug delivery systems similar to the one analyzed in this work, particularly because graphene oxide is typically used instead of graphene.

### 2.3. Cytotoxicity

**Figure 7:**
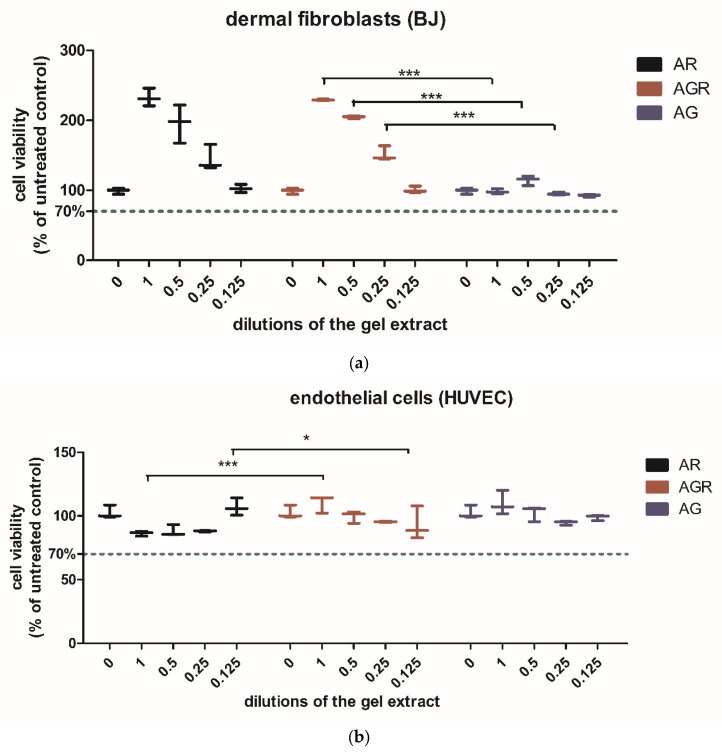
The effects of the gel extracts on the cell viability of dermal fibroblasts (BJ) (**a**), respectively endothelial cells (HUVEC) (**b**). Data is expressed as % of the untreated control, *n* = 3 (mean +/− SD). AR = alginate with resveratrol, AGR = alginate with resveratrol and graphene, AG = alginate with graphene. *** = *p* < 0.0001, * = *p* < 0.01 between the indicated groups.

As seen in Figure 7a,b, the dermal fibroblast proliferation was stimulated in the presence of the AR and AGR extracts in a dose-related manner. AG gel had little influence on the fibroblast’s proliferation; the 0.5 diluted extract had a slight stimulatory effect. Two-Way ANOVA showed significant overall differences between the experimental groups (*p* < 0.0001). The gel type and the concentrations used induced significantly different results (*p* < 0.0001).

In endothelial cells, the AR gel extract induced a decrease in viability without toxicity when used undiluted up to 0.25 dilution and had a slight proliferative effect in the lowest dilution (0.125). AGR gel extract induced a dose-dependent proliferation of the endothelial cells, but to a much lesser extent than in the fibroblasts. A slight proliferation was also induced by the AG gel extract in the undiluted and 0.5 diluted gel extract. Two-Way ANOVA showed significant overall differences between the experimental groups (*p* < 0.0001). The gel type (*p* < 0.001) and the concentrations used (*p* < 0.01) induced significantly different results, the concentration had a lower significance effect.

**Figure 8:**
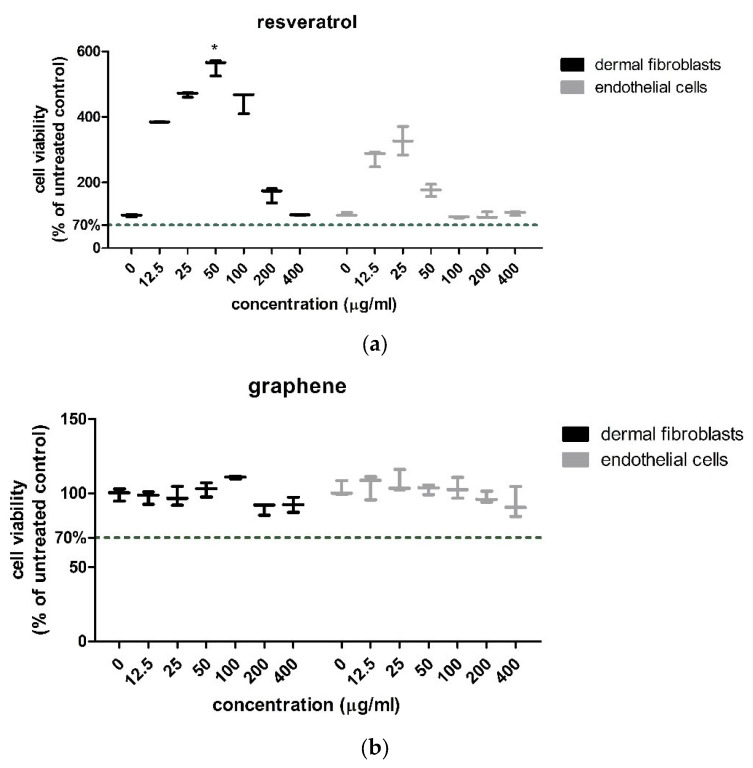
The effects of resveratrol (**a**) and graphene (**b**) on the cell viability of dermal fibroblasts (BJ), respectively, endothelial cells (HUVEC). Data are expressed as % of the untreated control, *n* = 3 (mean +/− SD). * = *p* < 0.01, compared to the control group.

The effects of resveratrol were stimulatory in both cell lines at lower doses. The effect was dose-dependent in the fibroblasts within the range of 12.5–25.0 µg/mL. Higher concentrations induced a lower proliferation, or none (at 400 µg/mL), but did not induce toxicity (Figure 8a). A similar effect, but less important, was induced by resveratrol in the endothelial cells. In both cultures, the most enhanced proliferation was obtained with the 25 µg/mL resveratrol. Kruskal–Wallis nonparametric test showed significant differences in the viability of the fibroblasts (*p* = 0.0041) and, respectively, endothelial cells (*p* = 0.0078) treated with different resveratrol concentrations.

To see if the graphene was well tolerated by the cells, we also checked for viability alterations in the presence of different graphene concentrations (Figure 8b). There was no toxicity in either of the cell lines tested up to 400 µg/mL graphene concentration. The fibroblasts showed a slightly enhanced proliferation at the concentrations of 50 and 100 µg/mL of graphene, while at the higher concentrations, viability was slightly decreased, but to around 90% of the control value. In the endothelial cells, the proliferation was increased in the presence of the graphene in concentrations between 12.5–50.0 µg/mL; higher concentrations diminished it to around 90% of the control. Overall, the graphene had a small effect on the cell’s viability. Kruskal–Wallis test showed no statistically significant differences in the fibroblasts or endothelial cell viability following graphene treatment.

## 3. Conclusions

In the present study, the system natrium alginate—graphene nanoplateles was prepared, characterized, and tested for resveratrol delivery. The release tests were conducted at physiological temperature (37 °C) for both AR and AGR. The amount of resveratrol released by AGR was greater than that released by AR at both tested pH (6.8 and 7.4). At pH 7.4, the delta remains positive throughout the entire experiment, meaning that the AGR sample consistently delivered a higher amount of the drug.

The natrium–alginate–graphene nanoplatelets were also very well tolerated by the normal human cells in vitro(fibroblasts and endothelial cells) and increased the proliferation of the cells in a concentration-dependent manner. Graphene had a minimal effect on the cells’ viability, depending on the concentration, without toxicity in either of the cell lines up to a concentration of 400 µg/mL.

Future research should consider conducting drug release tests over a longer period, beyond 24 h, and more extensive biological tests to confirm the promising properties of the natrium–alginate–graphene nanoplatelets system to function as a drug delivery system.

## 4. Materials and Methods

### 4.1. Materials

The gel was made from sodium alginate (NaAlg, M/G = 1.49; molecular weight = 396 kDa), graphene nanoplates (GNP), resveratrol, and CaCl_2_. The GNP was produced from commercially available intercalated graphite (Asbury Carbons, Anthracite Industries, Inc., Asbury, IA, USA) by microwave irradiation. Resveratrol was sourced from Sigma-Aldrich (St. Louis, MO, USA), and CaCl_2_ anhydrous was purchased from Chimreactiv S.R.L., Neamt, Romania. The resveratrol release medium consisted of phosphate-buffered saline (PBS, Merck, Germany, 1 tablet/200 mL).

#### 4.1.1. Synthesis of GNP

To obtain GNP, a method previously developed at the INFN NEXT Nanotechnology Laboratory in Frascati (Rome, Italy) was employed [34,45]. Firstly, graphite is put through microwave irradiation at 800 W 10 times until the particles develop a worm-like resemblance. Lastly, the product is exfoliated in isopropyl alcohol through pulsed-mode sonication at room temperature. Then, the alcohol is evaporated, and GNP flakes are obtained.

#### 4.1.2. Beads Preparation

For the experiments, two samples were prepared: the first consisting of alginate and resveratrol (AR) and the second consisting of alginate, resveratrol, and graphene. The polymer solution (A) was prepared by dissolving 400 mg of sodium alginate in 20 mL of water, and it was left under stirring for 20 min at room temperature. Meanwhile, the graphene nanoplatelet suspension (G) was prepared by dispersing 10 mg of GNP in 3 mL of isopropyl alcohol. The mixture was sonicated and then evaporated, followed by the addition of 10 mL of water. The suspension was sonicated again to achieve complete dispersion of the GNPs. A third solution (R) was prepared by dissolving resveratrol in a mixture of ethanol and water.

For the AR sample, 35 mg of resveratrol was dissolved in 0.5 mL of ethanol and 4.5 mL of distilled water and then added to solution A. For the AGR sample, 49 mg of resveratrol was dissolved in 0.5 mL of ethanol and 4.5 mL of distilled water and then added to the GNP dispersion. Finally, this mixture was added to solution A. The final concentrations in both AR and AGR were 0.28 mg/mL for GNPs and 1.4 mg/mL for resveratrol. To prevent resveratrol degradation, the solutions were kept in the dark.

Both AR and AGR solutions were left to rest for 10 min, after which they were drawn into syringes and slowly dripped into 200 mL of CaCl_2_ solution (0.1 M). The CaCl_2_ solution was prepared by dissolving 5.55 g of anhydrous CaCl_2_ in 500 mL of distilled water in a volumetric flask. When the solutions came into contact with the CaCl_2_ solution, small alginate beads were immediately formed due to the “egg-box” structure formed between Ca²⁺ ions and the carboxyl groups of the alginate. The strength of the gel beads is directly proportional to the concentration of the CaCl_2_ solution. The beads were left under stirring for 20 min to stabilize, then washed three times with distilled water. Finally, the beads were dehydrated in an oven at 65 °C.

### 4.2. Methods

#### 4.2.1. Characterization

##### UV-Vis Analysis

The quantification of resveratrol was performed on the release solutions using the UV-Vis spectrophotometer Shimadzu UV-1800, Kyoto, Japan. Calibration curves for pure resveratrol in PBS at pH 7.4 and pH 6.8 were obtained before the experiments to calculate the concentration of resveratrol released by the beads.

##### SEM

For morphological characterization of the beads, the scanning electron microscope (SEM) Hitachi SU8230, Tokyo, Japan, cold field emission operated at 30 kV, was used. The samples were mounted on brass supports with the help of a double-adhesive carbon tape.

For a better reflection of the electrons on the surface of the samples, they were covered with a 10 nm layer of platinum using the Agar Auto Sputter Coater, Rotherham, UK. Images were taken at different magnifications. Also, the images in Figure 1 were taken with an Olympus SZ stereo loupe, Tokyo, Japan.

##### Fourier Transform Infrared (FT-IR) Spectroscopy Analysis

FTIR spectra were recorded with a JASCO FTIR-6100 spectrometer (JASCO International Co., Ltd., Tokyo, Japan) over the spectral range of 4000 to 400 cm^−1^, with 4 cm^−1^ resolution using the KBr pellet technique. The pellets were prepared by mixing approximately 300 mg of anhydrous KBr with the dispersed sample in an agate mortar, followed by pressing the mixture into an evacuated die. The spectral data were collected and analyzed with the Jasco Spectra Manager v.2 software.

##### In Vitro Release Studies

The total amount of beads obtained was put in 20 mL of PBS pH 6.8 and 7.4 at 37 °C. The test tubes were covered with aluminum foil to keep the experiment in a dark environment. Aliquots of 0.8 mL of PBS were withdrawn every hour from the receiving medium, and an equal volume of fresh medium was added to maintain sink conditions. The calculations were performed using the following equation [46]:

(3) $$Q_{n}=C_{n}\times V_{0}+\sum_{i=1}^{n-1}C_{i}\times V.$$

Qn (mg) represents the cumulative release at the nth sampling point, Cn (mg/mL) is the concentration of the drug in PBS at the nth sampling point, V_0_ (mL) is the total volume of the release medium, Ci (mg/mL) is the concentration of the solution, and. V is the volume of each sample withdrawn. Various kinetic models (zero order, first order, Higuchi, Hixson–Crowell, and Korsmeyer–Peppas) were applied to fit the data obtained from the release tests.

##### Cytotoxicity Assay

Normal human cells, namely dermal fibroblasts (BJ-CRL-2522-ATCC, Gaithersburg, MD, USA) and human umbilical vein endothelial cells (HUVEC 2, Promocell, Hamburg, Germany) were used. Cells were cultivated in Dulbecco’s Modified Eagle Medium (DMEM), supplemented with 5% FCS for fibroblasts, respectively, human large vessel growth medium (Gibco, Invitrogen Waltham, MA, USA) both supplemented with penicillin, streptomycin, and amphotericin (Pan, Biotech GmbH. Aidenbach, Germany) in standard culture conditions. The medium was changed twice a week.

Cells (BJ and HUVEC) were cultivated on 96 well plaques at a density of 10^4^/well, accommodated for 24 h, and then exposed for 24 h to the hydrogel extracts and graphene and resveratrol in different concentrations ranging between 12.5–400 µg/mL. Gel extracts were obtained following ISO 10993-12:2012 proceedings [47]. Briefly, each gel sample (AGR, AG, and AR) was incubated in the fresh medium specific to each cell type at a concentration of 0.2 g/mL for 24 h at 37 °C. The gel samples were completely submerged in the medium. Medium extract was then collected and used immediately to treat the cells in different dilutions (undiluted, 0.5, 0.25, 0.125) made by using fresh medium. For resveratrol solubilization, we used DMSO at a concentration of 16 mg/mL; then, the stock solution was further diluted with PBS to obtain a stock solution of 4 mg/mL. This solution was immediately used to make the dilutions in the medium from the cell treatment. The final DMSO concentration was below 0.025%. A DMSO concentration below 0.1% is considered safe for almost all cells. Following the exposure, the toxicity of the compounds was measured by using the neutral red toxicology assay kit (TOX4 1KT, Sigma Aldrich, St. Louis, MO, USA), which stains viable cells, able to incorporate the dye into their lysosomes [48,49]. Samples were treated as indicated by the producer. Absorbance was red at 540 nm, using an ELISA plate reader. Cells treated with the medium were used as controls. All experiments were done in triplicate, and viability is expressed as % of untreated control.

##### Statistical Analysis

Statistical analysis of the significance of the cell viability differences in the experimental groups treated with graphene and resveratrol was assessed by the Kruskal–Wallis nonparametric test, followed by Dunn’s test. The statistical significance of the cell viability alterations induced by the gel type and concentration within the experimental groups treated with AR, AG, and AGR gels was tested by Two-Way ANOVA, followed by Bonferroni posttest, and using GraphPad Prism version 5.00 for Windows (GraphPad Software, San Diego, CA, USA, www.graphpad.com, accessed on 12 March 2007). *p* < 0.05 was considered significant. The drug release experiments were performed in triplicate for all samples to ensure statistical reliability. The data were analyzed using Microsoft Excel (version 2411, Microsoft Corp., Redmond, WA, USA). The standard deviation (SD) was calculated by the software, and the results were further analyzed for statistical significance using one-way analysis of variance (ANOVA), with a significance level set at *p* < 0.05.

## Figures and Tables

**Table 1:** Regression coefficient and kinetic parameters of the samples for the whole experiment.

Regression Coefficients (R^2^)					
Sample	Zero Order	First Order	Higuchi	Hixson-Crowell	Korsmeyer-Peppas
AR 6.8	0.7334	0.805	0.9424	0.783	0.9221
AGR 6.8	0.6658	0.8273	0.9091	0.784	0.8988
AR 7.4	0.7529	0.8191	0.9491	0.799	0.9282
AGR 7.4	0.6235	0.7484	0.892	0.709	0.8636
**Kinetic Parameters**					
Sample	Zero Order (k_0_)	First Order (k_1_)	Higuchi (K_H_)	Hixson-Crowell (t_f_)	Korsmeyer-Peppas (t^n^)
AR 6.8	4.655	−0.0249	0.0775	0.0826	0.0266
AGR 6.8	8.4852	−0.0581	0.0422	0.1768	0.0144
AR 7.4	4.4784	−0.0237	0.0817	0.0789	0.0282
AGR 7.4	6.4536	−0.039	0.0532	0.124	0.0176

**Table 2:** Regression coefficient and kinetic parameters of the samples for the first 6 h of the experiment.

Regression Coefficients (R^2^)					
Sample	Zero Order	First Order	Higuchi	Hixson-Crowell	Korsmeyer-Peppas
AR 6.8	0.8663	0.906	0.9906	0.894	0.8118
AGR 6.8	0.8566	0.9389	0.9879	0.9154	0.797
AR 7.4	0.8941	0.9263	0.9895	0.9163	0.8211
AGR 7.4	0.8044	0.8751	0.9697	0.8527	0.7419
**Kinetic Parameters**					
Sample	Zero Order (k_0_)	First Order (k_1_)	Higuchi (K_H_)	Hixson-Crowell (t_f_)	Kors-Peppas (t^n^)
AR 6.8	4.655	−0.0249	0.0775	0.0826	0.0266
AGR 6.8	84852	−0.0581	0.0422	0.1768	0.0144
AR 7.4	4.4784	−0.0237	0.0817	0.0789	0.0282
AGR 7.4	6.4536	−0.039	0.0532	0.124	0.0176

## Data Availability

The original contributions presented in this study are included in the article. Further inquiries can be directed to the corresponding author.
